# Pretraining Strategies for Structure Agnostic Material Property Prediction

**DOI:** 10.1021/acs.jcim.3c00919

**Published:** 2024-02-01

**Authors:** Hongshuo Huang, Rishikesh Magar, Amir Barati Farimani

**Affiliations:** † Department of Material Science and Engineering, Carnegie Mellon University, Pittsburgh, Pennsylvania 15213, United States; ‡ Department of Mechanical Engineering, Carnegie Mellon University, Pittsburgh, Pennsylvania 15213, United States

## Abstract

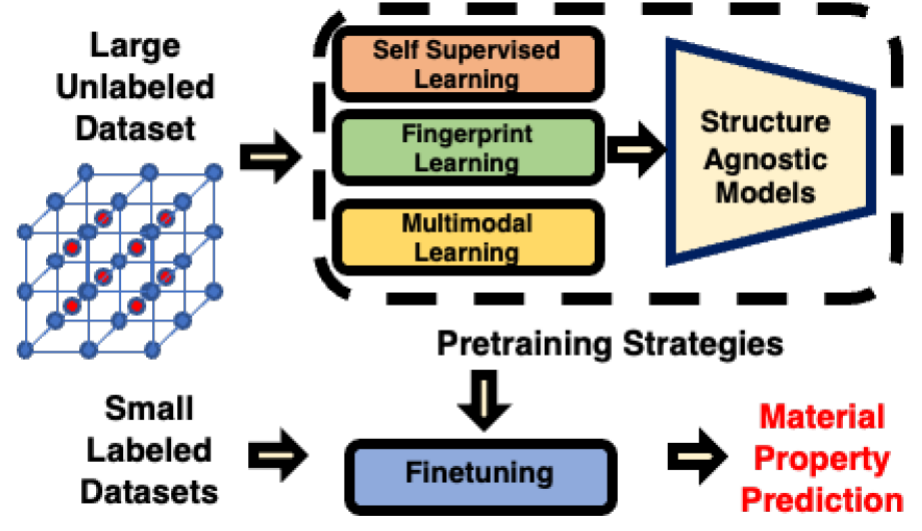

In recent years, machine learning (ML), especially graph neural network (GNN) models, has been successfully used for fast and accurate prediction of material properties. However, most ML models rely on relaxed crystal structures to develop descriptors for accurate predictions. Generating these relaxed crystal structures can be expensive and time-consuming, thus requiring an additional processing step for models that rely on them. To address this challenge, structure-agnostic methods have been developed, which use fixed-length descriptors engineered based on human knowledge about the material. However, the fixed-length descriptors are often hand-engineered and require extensive domain knowledge and generally are not used in the context of learnable models which are known to have a superior performance. Recent advancements have proposed learnable frameworks that can construct representations based on stoichiometry alone, allowing the flexibility of using deep learning frameworks as well as leveraging structure-agnostic learning. In this work, we propose three different pretraining strategies that can be used to pretrain these structure-agnostic, learnable frameworks to further improve the downstream material property prediction performance. We incorporate strategies such as self-supervised learning (SSL), fingerprint learning (FL), and multimodal learning (ML) and demonstrate their efficacy on downstream tasks for the Roost architecture, a popular structure-agnostic framework. Our results show significant improvement in small data sets and data efficiency in the larger data sets, underscoring the potential of our pretrain strategies that effectively leverage unlabeled data for accurate material property prediction.

## Introduction

**Figure 1:**
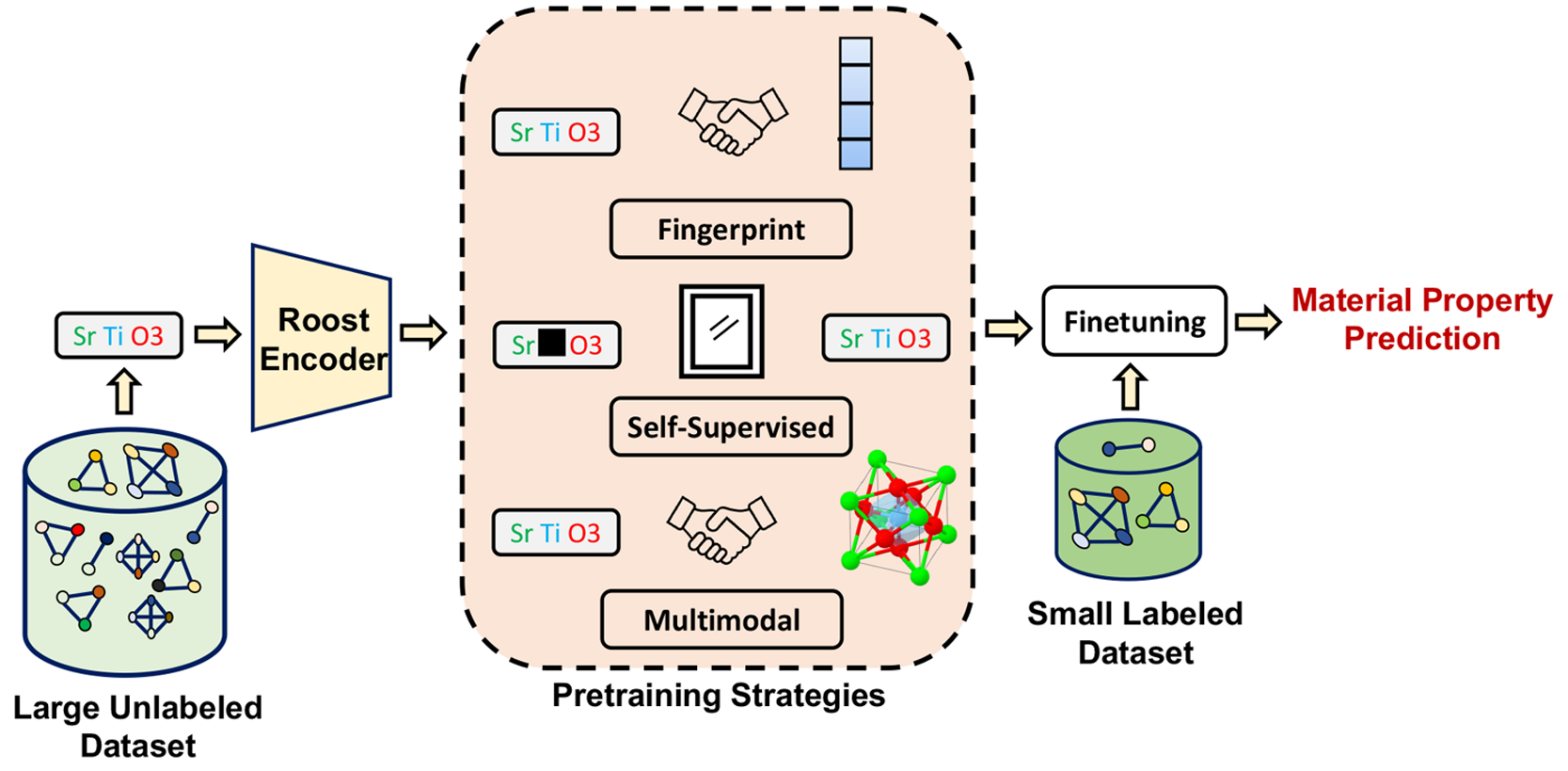
Framework for all the proposed pretraining strategies. We use the Roost encoder to demonstrate the effectiveness of the pretraining strategies for material property prediction tasks. We propose three strategies: 1.) Self-Supervised Learning 2.) Fingerprint Learning, and 3.) Multimodal Learning. Using these strategies we pretrain the Roost Encoder and finetune the model on different data sets in the Matbench^41^ suite. Using such pretraining strategies we are able to demonstrate improvements on downstream tasks.

Machine learning (ML) models have made significant progress in computational material science, both in material property prediction^1−10^ and new material generation.^11^ The growth of ML models in material science has been fueled by the increasing number of publicly available data sets and improved hardware capabilities.^12−14^ Popular ML frameworks take the crystalline structure as input and leverage graph neural networks (GNNs) to construct representations that can be used for property prediction. In these frameworks, the crystal structure information like 3D coordinates is required to construct a graph of the crystalline material.^9,15−18^ The general idea is to consider the atoms as the nodes and capture the interactions between them by using edges. This structure captures the interactions in the crystalline material, and the models often take optimized structures that are generated via simulations or experiments. Despite the large availability of crystal structures in public repositories such as Materials Project^19^ and ICSD,^20^ it only represents a fraction of the chemical space of materials. Generating the crystalline structures for all materials in the vast materials space can be a time-consuming process. This has motivated researchers to develop methods that do not require the structure of the material for crystals that do not have a well-defined structure beforehand. These structure agnostic methods can possibly be used for the high-throughput screening of materials with desired properties. The general approach to developing these structure agnostic models is using fixed length descriptors that encode the chemical composition of the material. These fixed length descriptors can be used to construct a feature vector that captures the material’s properties, which can be used to predict its behavior.^21−23^ However, the drawback of this approach is that these fixed length descriptors need to be handcrafted and require considerable domain knowledge and expertise. Recently, multiple approaches leveraging structure agnostic representations for material property predictions have been developed.^17,24−27^ In this work, we focus on the Representation Learning from Stoichiometry (Roost) framework proposed by Goodall et al.^26^ The Roost model takes as input the crystal formula and constructs a graph based representation to develop a learnable framework. The Roost architecture is able to predict the material properties with reasonable accuracy using only the stoichiometric data. In this work, we utilize the Roost model and propose three different pretraining strategies to improve the performance of the framework. Our pretraining strategies include 1.) Self Supervised Learning (SSL), 2.) Fingerprint Learning (FL), and 3.) Multimodal Learning (MML). After pretraining the Roost model with the 3 pretraining strategies, we observe performance gains in multiple material property prediction tasks (Figure 1).

For our first strategy, we propose the Self-Supervised Learning approach (SSL) for pretraining the Roost encoder. In recent years, SSL frameworks^28−36^ have been successfully utilized in computer vision and natural language processing tasks. The successful application of SSL has spurred many works in molecular machine learning^37−39^ and material science.^3,40^ Drawing upon the successful strategies of SSL employed in structure-based material property prediction,^3,7^ we propose a framework for structure-agnostic SSL using the Roost encoder for generating material representation. The core idea of SSL revolves around pretraining models without the reliance on explicitly labeled data sets by leveraging the intrinsic information present in unlabeled data as the training signal. This framework can especially be advantageous for structure-agnostic material property prediction tasks, where labeled data may be scarce and complete structural characterization of material is sometimes unavailable. By adopting this approach, we address the challenges associated with limited labeled data and inaccessible structural information. For the FL strategy, we devise a simple methodology of predicting the Magpie fingerprint^21^ using the Roost encoder; the core idea is that the pretrained model can learn the information captured by the fingerprint. Using such a strategy allows us to build a Roost encoder that can retain the benefits of being a learnable framework and also capture information on a fixed descriptor like the Magpie fingerprint. We also introduce a MML strategy in which we leverage the available characterized structure data and predict the embedding generated using a pretrained CGCNN^1^ encoder from the Crystal Twins Framework.^3^ Using such a strategy, we are able to learn the structural information using our structure agnostic encoder. By incorporating these three strategies, we successfully enhance the performance of the Roost encoder in downstream tasks within the Matbench suite. Notably, we demonstrate improvements in most material property prediction tasks, highlighting the effectiveness and potential of our proposed pretraining strategies.

## Methods

In this section, we describe the pretraining strategies and the ROOST encoder that we use to demonstrate the efficacy of the pretraining strategies. We introduce three pretraining strategies in this work: 1) Self-Supervised Learning (SSL), 2) Fingerprint Learning (FL), and 3) Multimodal Learning (MML). For pretraining, we leverage an unlabeled large data set to train the Roost model. The success of pretraining strategies depends largely on the quantity and quality of the pretraining data set.

### Pretraining and Finetuning Data Sets

The performance of downstream tasks in pretraining models is heavily influenced by both the quality and quantity of the pretraining data. To assess the impact of pretraining data, we consider two perspectives: data quantity and data quality. In our work, we utilize three distinct groups of data: 1) the data set used in Roost (OQMD and mp-nonmetal-band gap) consisting of 304,433 entries, 2) a combined data set from Matbench comprising 408,065 data points, and 3) a set of 137,652 MOF data. We determine that a pretraining data set size of 432,314 data points, obtained by selecting the unique combination from all three data sets, gives us maximum improvements on downstream tasks (Table S4). Additional details can be found in the Supporting Information. The finetuning data sets aggregated from Matbench^41^ to evaluate the performance of the pretrained models are shown in Table 1. We select 9 different data sets with a diverse range of properties.

**Table 1:** Overview of the Data Sets Used for Benchmarking the Performance of the Pretraining Framework^a^

Data set	# Samples	Property	Unit
Steelds^41^	312	Yield Strength	MPa
JDFT2D (JDFT)^42^	636	Exfoliation Energy	meV per atom
Phonons^43^	1,265	Last Phdos Peak	1 per cm
Dielectric^44^	4,764	Refractive Index	Unitless
GVRH^45,46^	10,987	Shear Modulus	log_10_ GPa
KVRH^46^	10,987	Bulk Modulus	log_10_ GPa
Perovskites^47^	18,928	Formation Energy	eV per atom
MP-Gap (MP-BG)^19^	106,113	Band Gap	eV
MP-E-Form (MP-FE)^19^	132,752	Formation Energy	eV per atom

a We predict the properties of 9 different data sets^19,42−49^ aggregated from the Matbench suite.^41^

### Structure Agnostic Representation Encoder

**Figure 2:**
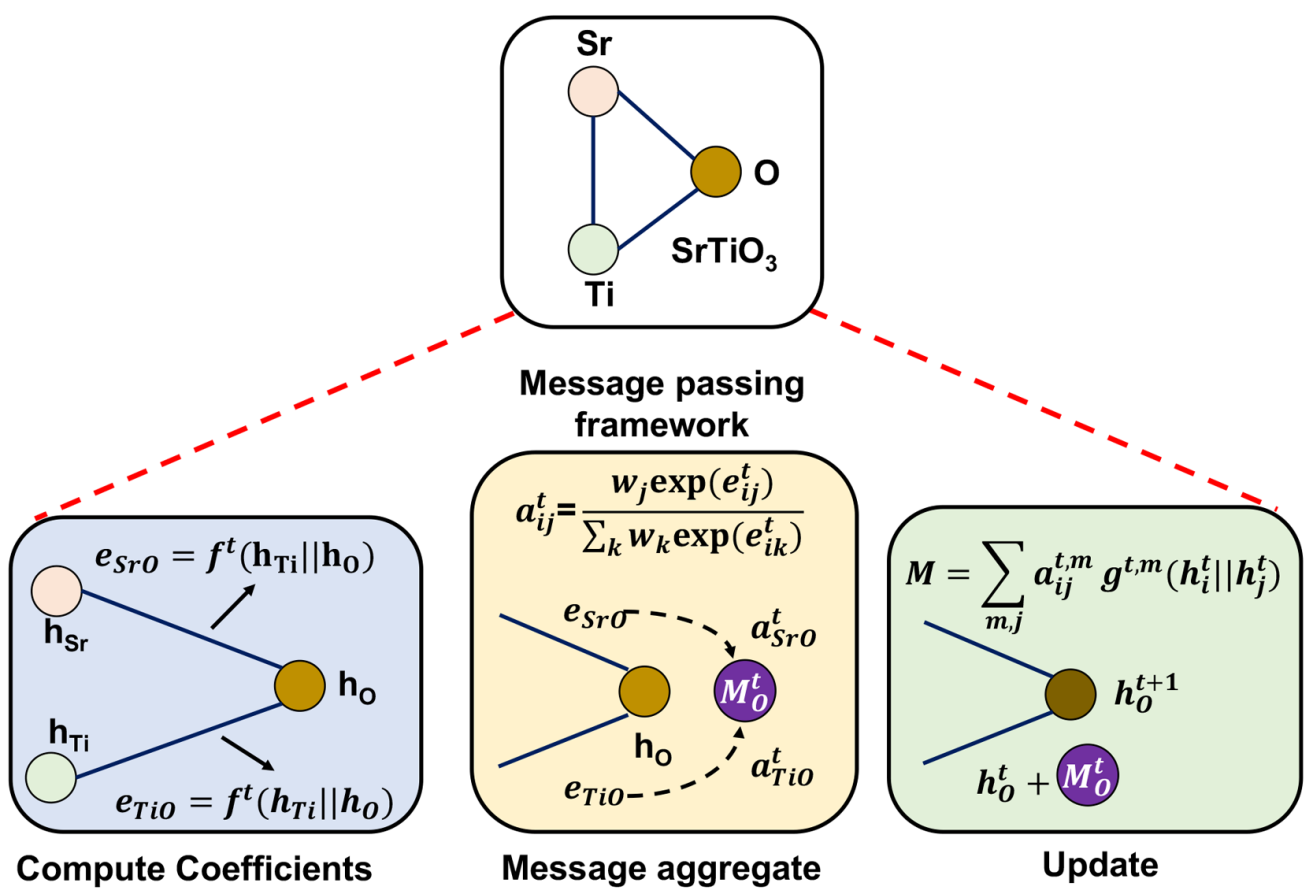
Roost model utilizes the stoichiometric formula as input, such as SrTiO_3_, to create a graph representation of the material. The figure demonstrates the message passing specifically for node *O*; the node update process for all nodes is the same. The message passing framework in Roost consists of three key components. First, unnormalized scalar coefficients are computed for each edge in the graph. These coefficients are then normalized in the message aggregation step using soft attention, allowing for the aggregation of messages from all connected nodes. Finally, in the update step, the node representations are updated in a residual manner.^51^

To generate the structure agnostic representation, we use the ROOST encoder. The ROOST encoder takes as input the stoichiometric formulas which are used to construct a dense weighted graph coupled with a message-passing framework to learn material descriptors. The dense weighted graph consists of all of the elements in the stoichiometric formula. For example, consider the stoichiometric formula SrTiO_3_ shown in Figure 2. This formula contains three unique elements: Sr, Ti, and O; a fully connected weighted graph is formed with nodes representing these elements. The initial element (node) representations are generated from the Matscholar embeddings^50^ which are then multiplied by a learnable weight matrix to generate the internal representation for the message passing framework. The Roost architecture is illustrated in Figure 2.

The message-passing framework in Roost updates the node information in multiple stages, as represented in Figure 2. The first step is to calculate eq 1
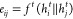
1where *e*_*ij*_ is the unnormalized scalar coefficient for a pair of nodes, and ∥ is the concatenation operation. *f*^*t*^ is a multilayer perceptron (MLP), and *h*_*i*_^*t*^ is the node feature for the central atom whose node embedding is currently being updated. *h*_*j*_^*t*^ represents the neighbor embedding with index *j* running over all the neighbor elements in the graph. In the second step, the scalar coefficient *e*_*ij*_ is then normalized using a weighted softmax function as shown in eq 2
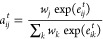
2where *w*_*j*_ is the fractional weight of the elements in the composition, *j* is the current neighbor atom, and *k* includes all the neighbor atoms. Finally, the update of the node features happens using skip connections,^51^ and the update equation is given by eq 3
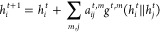
3where *g*^*t*,*m*^ is an MLP, and *h*^*t*+1^ represents the current update node feature after the *t*^*th*^ layer. Following the approach of the Roost paper,^26^ we employ a weighted attention pooling-based operation. This technique considers each element in the weighted graph and allocates attention to them based on their learned representations. The resulting pooled representation is then fed into a multilayer perceptron (MLP) to make the final material property prediction.

### Self-Supervised Learning

**Figure 3:**
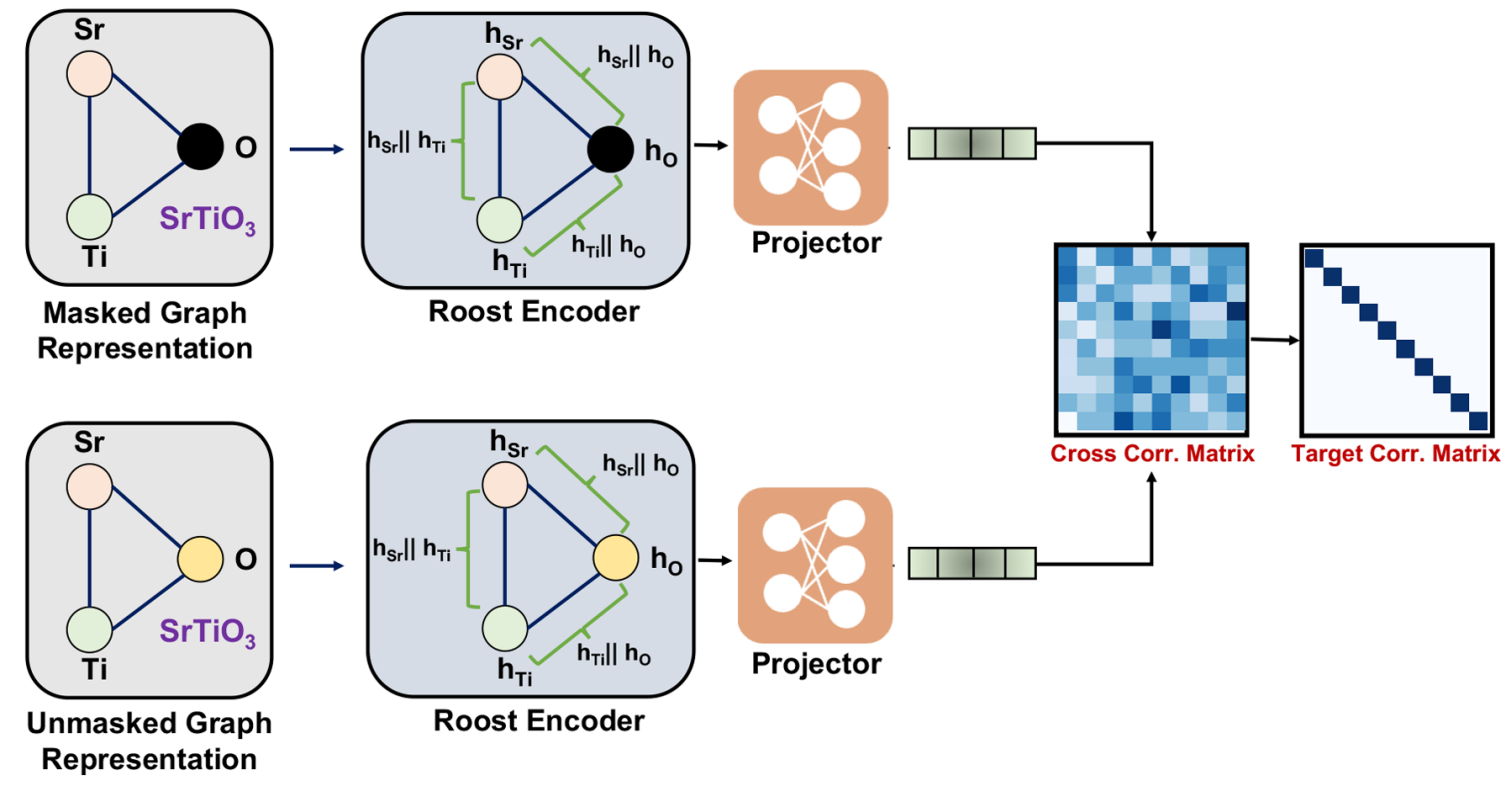
We develop a self-supervised learning based framework for the Roost encoder.^26^ We use the Barlow Twins Framework^30^ for pretraining the Roost model. Two different augmentations are created and fed to the Roost Encoder. The goal of pretraining is to push the empirical cross-correlation matrix to the identity matrix.

For Self-Supervised Learning (SSL), we use the Barlow Twins framework introduced by Zbontar et al.^30,52^ The main idea is to create two different augmentations from the same crystalline material^53^ and to make the encoder representations for these augmentations as similar to each other as possible (see Figure 3). The augmentation technique that we implemented was random atom masking. In this technique, we masked 10% of the nodes in the formula graph; if 10% of the nodes were less than one, a default of one masked atom was applied. The objective of the pretraining stage is to push the empirical cross-correlation matrix, generated from the encoder representations of the unmasked and masked graphs, toward the identity matrix. The cross correlation matrix is formulated by using the embeddings generated from the enhancements of the same material. The values in the cross correlation matrix range between −1 to 1. The formula to calculate the elements in the cross correlation matrix is shown in eq 4
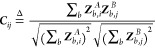
4

Compared with other SSL methodologies such as contrastive learning, the Barlow Twins loss does not explicitly require positive and negative pairs for pretraining. The mathematical representation of the Barlow Twins loss function is given by eq 5
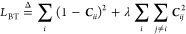
5where ***C*** represents the cross-correlation matrix of embeddings from two augmented instances. ***Z***^*A*^ and ***Z***^*B*^ are the projection embedding from the masked and unmasked graph representations, *b* indexes the batch sample, and *ij* index the vector dimension. The hyperparameters for the SSL pretraining technique are given in Table S1.

### Fingerprint Learning

**Figure 4:**
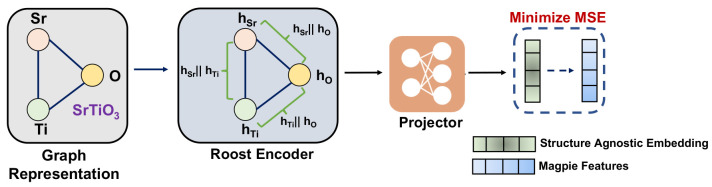
In the Fingerprint Learning strategy, we use the Roost encoder^26^ to predict the Magpie Fingerprint.^21^ Using such a strategy allows our framework to capture the features from the fixed length descriptors helping to improve downstream prediction performance.

For Fingerprint Learning (FL), we utilized the Magpie feature^21^ as the fingerprint to represent the materials. It offers a broad range of physical/chemical properties of 145 features, including Stoichiometric attributes, which depend only on the fractions of elements present and several *L*^*p*^ norms of the fractions; Elemental property statistics, which consist of mean, mean absolute, deviation, range, maximum, minimum, and mode of 22 element properties, including rows in the Periodic table, atomic number, and atomic radii; Electronic structure attributes, the average fraction of electrons from *s*, *p*, *d*, and *f* valence shells; Ionic compound attributes including features determine whether the elements could form an ionic compound. By utilizing the Magpie^21^ feature set, we can capture a diverse array of material properties. We predict the Magpie fingerprint^21^ using the Roost encoder. The loss function we used for FL is given by eq 6 as the mean square error between the fingerprint and the embedding from the Roost encoder
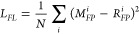
6where *L*_*FL*_ is the fingerprint loss, *M*_*FP*_^*i*^ is the Magpie Fingerprint for the *i*^*th*^ sample, and *R*_*FP*_^*i*^ is the embedding generated from the Roost Encoder and projector, and we have *N* total samples. As shown in Figure 4, the employed projector is a simple linear layer, which functions to align the roost embedding with the dimensional space of the Magpie Descriptor. The hyperparameters for the FL strategy are given in Table S2.

### Multimodal Learning

The Multimodal Learning (MML) strategy integrates both structure-agnostic and structure-based approaches, specifically focusing on how structure embeddings can be used to pretrain structure-agnostic encoders. While structure-based graph neural networks are generally more accurate due to their ability to capture local environments, the MML framework aims to explore whether a low-cost structure-agnostic model can mimic some of the features captured by structure-based models. By leveraging pretrained models like Crystal Twins^3^ on structural data, we can generate embeddings for data sets unrelated to the finetuning benchmarks and utilize the structure-agnostic encoder to predict these embeddings.

In this work, we use the hMOF database^3^ to generate structure embeddings using a pretrained Crystal Twins model, and these embeddings are then predicted using the Roost encoder. The choice of hMOF as the structure data set was deliberate, as it is unrelated to the downstream finetuning tasks, allowing us to decouple the influence of structure-based models on the Roost encoder during finetuning. This approach was adopted to thoroughly examine the potential impact of such a strategy. The loss function utilized for pretraining the model is presented in eq 7
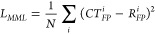
7where *CT*_*FP*_^*i*^ is the embedding for the *i*^*th*^ sample generated by the Crystal Twins encoder, and *R*_*FP*_^*i*^ is the embedding generated from the Roost Encoder and projector, and we have *N* total samples. Similar to the FL, here, we employed a linear layer to project the roost embedding to match the dimension of the CGCNN embedding. The hyperparameters for the MML strategy are given in Table S3.

## Results

### Performance on Matbench

To evaluate the effectiveness of the pretraining strategies, we use the 9 materials property prediction data sets from MatBench^41^ as the downstream tasks. These data sets cover a diverse range of properties, including yield strength of steels (MPa), exfoliation energy (meV/atom), frequency of the highest frequency optical phonon mode peak (cm^–1^), refractive index (*n*), bulk modulus (log_10_(GPa)), shear modulus (log_10_(GPa)), formation energy of perovskite (meV/unit cell), bandgap energy (eV), and formation energy (eV/atom).

To assess the performance of the pretraining strategies and ensure a fair comparison, we followed the 5-fold nested cross-validation protocol of the Matbench benchmark for each data set. Our pretrained model was compared with the Roost model, which utilized the same hyperparameters (Table S6). Additionally, we compared the results with three other structure-agnostic models: Finder,^17^ AtomSets,^25^ and CrabNet.^24^ We also add a conventional machine learning baseline that uses Magpie Fingerprints coupled to random forest (RF-Magpie). The comparative results are presented in Table 2.

**Table 2:** MAEs of Different Structure-Agnostic Models for Predicting Matbench Benchmark Properties^a^

Data sets	Finder	AtomSets	CrabNet	Roost	Roost-SSL	Roost-FL	Roost-MML	RF-Magpie
Steels^41^	-	-	-	130 ± 20.30	111 ± 5.7	130 ± 33.6	126 ± 17.3	**102** ± 11.6
JDFT^42^	48	52	45.6	44.64 ± 11.73	42.87 ± 11.93	**42.65** ± 12.35	45.00 ± 13.37	50.57 ± 9.1
Phonons^43^	46.6	63	55.1	54.38 ± 4.73	**46.05** ± 4.22	51.93 ± 6.95	53.33 ± 5.61	64.72 ± 9.31
Dielectric^44^	0.3204	0.36	0.3234	0.3252 ± 0.0780	**0.3122** ± 0.0808	0.3167 ± 0.0779	0.3221 ± 0.0761	0.4204 ± 0.0783
GVRH^46^	**0.0996**	0.11	0.1014	0.1034 ± 0.0020	0.1006 ± 0.0023	0.1046 ± 0.0029	0.1032 ± 0.0021	0.1049 ± 0.0019
KVRH^46^	0.0764	0.08	**0.0758**	0.0797 ± 0.0042	0.0777 ± 0.0041	0.0776 ± 0.0031	0.0782 ± 0.0047	0.0825 ± 0.0033
Perovskites^47^	0.645	**0.082**	0.407	0.4025 ± 0.0077	0.4050 ± 0.0086	0.4043 ± 0.0091	0.4013 ± 0.0077	0.5809 ± 0.0109
MP-BG^19^	**0.231**	0.26	0.266	0.2571 ± 0.0055	0.2646 ± 0.0041	0.2560 ± 0.0037	0.2551 ± 0.0104	0.2657 ± 0.0013
MP-FE^19^	**0.0839**	0.094	0.0862	0.0847 ± 0.0016	0.0854 ± 0.0012	0.0843 ± 0.0017	0.0834 ± 0.0010	0.1167 ± 0.0013

a We also compare the pretrained models with other supervised learning baselines. The best performing result is shown in boldface, and next best performing result is underlined.

Our Roost-SSL model demonstrated improvements in 6 out of the 9 data sets over the supervised Roost model, with significant enhancements observed in the smaller data sets (JDFT, Phonons Dielectric, and Steels). The average improvement for the smaller data sets was 10.21% (Table S5). Meanwhile, the Roost-FL model showed improvements in 6 out of the 9 data sets, and the Roost-MML shows improvements for 7 out of 9 data sets over the supervised Roost model. The improvements for Roost-SSL are more pronounced enabling it to achieve the best performance among the benchmarked models on 3 out of the 9 data sets (Table S5).

When compared to the other structure agnostic models like Finder,^17^ AtomSets,^25^ CrabNet^24^ and Roost,^26^ our pretrained models exhibited superior performance for smaller data sets and similar performance for medium-sized data sets. The performance for the larger data sets, however, was poorer for Roost-SSL and Roost-FL models. The reason behind this is that the larger data sets such as band gap and formation energy data sets contain 106,113 data points and 132,752 data points, respectively, which is significantly larger than the small data sets with only 636 (JDFT) to 10,987 (GVRH) data points, allowing supervised learning to achieve good performance because the effect of pretraining is not prominent for larger data sets. For the Roost-MML strategy, we are able to improve upon the performance of the baseline Roost model for larger data sets. This is possibly due to the model trained with MML strategy can capture some of the structure based features, and it may have helped in enhancing the downstream performance. It must be noted that during pretraining for the MML strategy we pretrained on the hMOF data set which is unrelated to the downstream benchmarks. While our approach aims to integrate out-of-distribution structural information into the structure-agnostic GNN, we observed that this does not consistently yield improved performance across all tasks. This limitation may partly stem from the fact that we used hMOF to generate CGCNN embeddings used for pretraining with MML. The hMOF data set encompasses only 11 elements, and since the Roost Encoder utilizes elemental information as nodes, the limited diversity within the pretraining data set could be another factor constraining performance enhancement. Despite these limitations, the observed performance improvements in certain downstream tasks suggest that structure-agnostic models, particularly when employing the MML strategy, have the potential to approximate the embeddings of structure-based models effectively.

**Figure 5:**
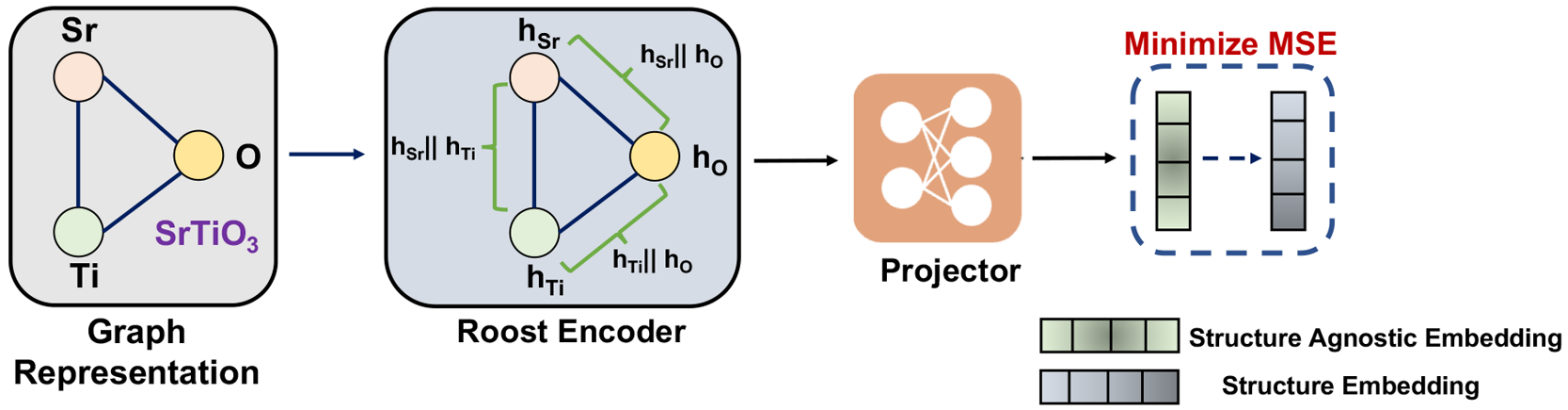
In the Multimodal Learning strategy, we use the Roost encoder^26^ to predict the Structure Based Embedding from the Roost encoder.^3^ Using such a strategy allows our framework to capture the features from the structure based embeddings helping to improve downstream prediction performance.

Overall, the improvements demonstrated using the three proposed pretraining strategies suggest that they can be effective especially in low data regimes enabling the structure agnostic model to act as screening tools especially in cases where structure data are not available for materials (see Figure 5).

### Analyzing the Pretrained Representations

To assess the effectiveness of various pretraining strategies, we combined pretrained embeddings with a random forest regressor, keeping the encoder parameters frozen. The results of the ablation are shown in Table 3. We examined all three pretraining methods: Self-Supervised Learning (SSL), Fingerprint Learning (FL), and Multimodal Learning (MML). These were compared to a baseline model that employs one-hot embeddings, which are used as node representations by several GNNs such as CGCNN,^1^ OGCNN,^2^ and ALIGNN.^54^ We compute the one-hot embeddings for all elements in the stoichiometry and then apply one forward pass. This processed data are then passed to the random forest, which we refer to as the Baseline. Compared to the one-hot embedding baseline, all three pretraining methods outperformed in most data sets, with the fingerprint learning strategy taking the lead in downstream tasks. This might be because fingerprint learning can incorporate certain chemical knowledge from Magpie fingerprints. In general, the performance of the pretrained embeddings, when compared to the baseline, suggests that the model captures chemical knowledge during the pretraining phase. The SSL and FL strategy outperforms the baseline in 8 out of 9 data sets and has comparble performance on the steels data sets. The performances of the pretrained model and the baseline are considered comparable if they are within the standard deviation. The MML strategy outperforms the baseline on phonons and shows comparable performance on JDFT and Dielectric data sets. One of the possible reasons for this poor performance may be that the pretrained hMOF data set only contains 11 elements. We also note that the performance of pretrained embeddings with respect to the Magpie fingerprint coupled with random forest (RF-Magpie) is poor; however, after finetuning, the performance of all the pretrained models is better than RF-Magpie (Table 2).

**Table 3:** Random Forest with Pretrained Embeddings Compared to the One-Hot Baseline and Dummy^a^

Data set	Roost-SSL	Roost-FL	Roost-MML	Baseline	Dummy
Steels^41^	130 ± 16.9	119 ± 16.8	143 ± 21.1	**100** ± **19.01**	230 ± 9.7
JDFT^42^	60.56 ± 10.95	**59.15** ± **7.94**	66.64 ± 10.33	61.51 ± 6.88	67.29 ± 10.18
Phonons^43^	166.34 ± 14.58	**97.11** ± **22.00**	193.29 ± 10.53	234.78 ± 17.69	323.98 ± 17.73
Dielectric^44^	0.5395 ± 0.0658	**0.4425** ± **0.0691**	0.5939 ± 0.0728	0.5685 ± 0.0526	0.8088 ± 0.0718
GVRH^46^	0.1538 ± 0.0015	**0.1221** ± **0.0020**	0.2171 ± 0.0028	0.1994 ± 0.0035	0.2931 ± 0.0031
KVRH^46^	0.1376 ± 0.0027	**0.1003** ± **0.0024**	0.2055 ± 0.0042	0.1615 ± 0.0023	0.2897 ± 0.0043
Perovskites^47^	0.5940 ± 0.0092	**0.5868** ± **0.0109**	0.6200 ± 0.0088	0.5958 ± 0.0113	0.6450 ± 0.0167
MP-BG^19^	0.5187 ± 0.0478	**0.3942** ± **0.0041**	0.6476 ± 0.0049	0.5775 ± 0.0062	1.3272 ± 0.006
MP-FE^19^	0.2590 ± 0.0014	**0.1459** ± **0.0010**	0.3375 ± 0.0009	0.2776 ± 0.0020	1.0059 ± 0.003

a The Dummy baseline uses the mean prediction strategy. The best performing result is shown in boldface, and next best performing result is underlined.

**Figure 6:**
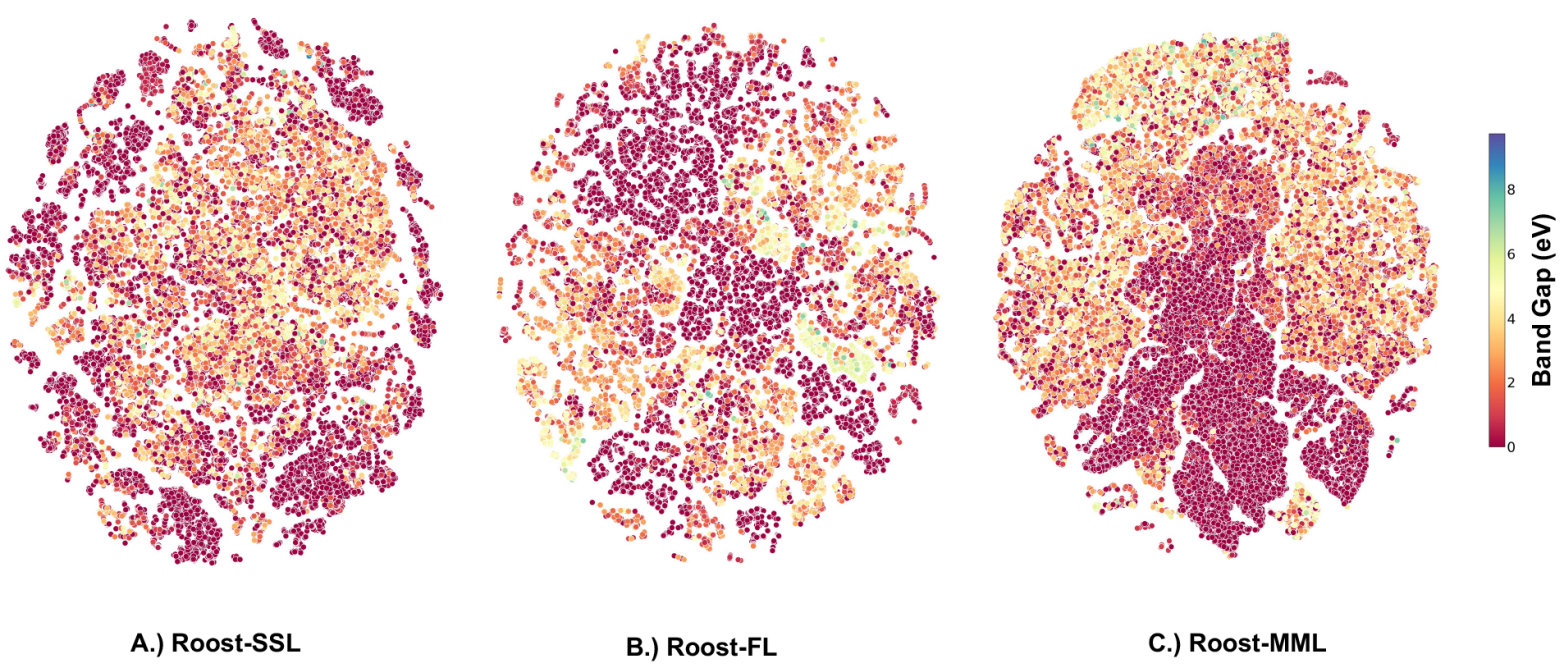
t-SNE representation for the band gap data set. A.) Pretrained Roost-SSL representation for the band gap data set. B.) Pretrained Roost-FL representation for the band gap data set. C.) Pretrained Roost-MML representation for the band gap data set.

Furthermore, we examined the pretrained representations learned by the model for the band gap and the formation energy data set using t-SNE.^55^ These representations were captured before fine-tuning the model on the specific data sets. For the band gap data set, the t-SNE representation is shown in Figure 6, with data samples colored according to their band gap value. In the Roost-SSL representation (Figure 6A), low band gap samples can be seen scattered around the plot’s edges. For the Roost-FL strategy, these low band gap samples cluster at the top left and centrally (Figure 6B). With the Roost-MML (Figure 6C) strategy, the clustering appears more distinct than the other methods, aligning with its superior performance in the downstream task of band gap prediction. In this case, samples with low band gap are located at the bottom right, while those with higher values occupy the top region of the plot.

**Figure 7:**
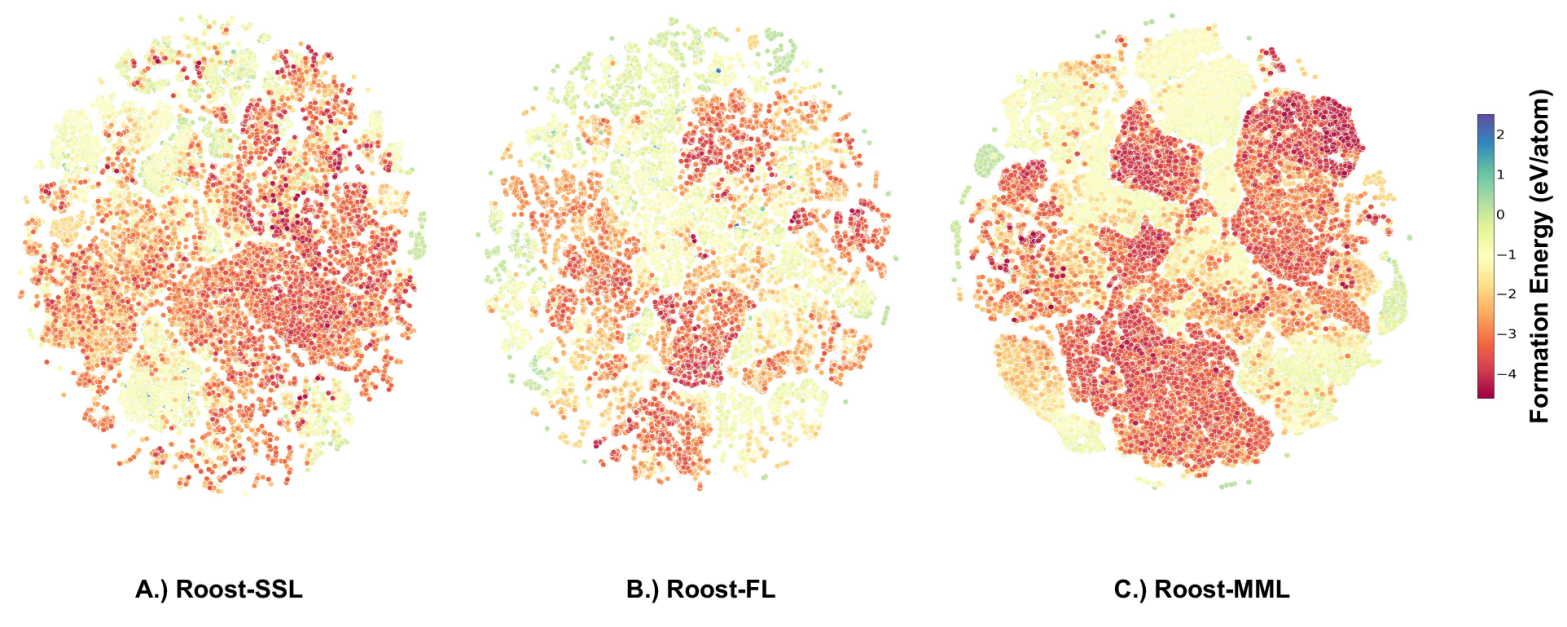
t-SNE representation for the formation energy data set. A.) Pretrained Roost-SSL representation for the formation energy data set. B.) Pretrained Roost-FL representation for the formation energy data set. C.) Pretrained Roost-MML representation for the formation energy data set.

The t-SNE representations for the formation energy data set are shown in Figure 7. When visualizing the representation for the formation energy data set, there is a noticeable clustering, especially with the MML strategy. Materials with low formation energy cluster together on the right side of the plot (Figure 7C). Materials with high formation energy are centrally located in the fingerprint learning strategy plot (Figure 7B) and lean toward the right in the SSL strategy plot (Figure 7A). These observations suggest that pretrained models can effectively encode the underlying chemistry, which could enhance downstream performance.

### Small Data Set Utility of Pretraining

Our observations from the downstream performance on Matbench revealed that both the Roost-SSL and Roost-FL strategies were particularly effective for smaller data sets (those with fewer than 5000 samples). This has potential implications for new material discovery as initial data sets for new material discovery are generally small, so having models that can perform well on such data sets can be highly beneficial.

To determine the statistical significance of the results, we used a paired *t* test. The outcomes for statistical significance are presented in Table 4. A negative t-statistic suggests that the MAE of the pretrained model is less than that of the baseline Roost model. The magnitude (in absolute value) of the t-statistic reflects the strength of the observed mean difference in MAE between the pretrained and baseline models relative to the variability between the paired observations for both models. If the p-value is below 0.05, it indicates that the results are statistically significant, meaning that the improvement achieved through pretraining is significant.

**Table 4:** Statistical Significance of the Results for the Smaller Data Sets (<5000 Samples)^a^

Data set	Roost-SSL	Roost-FL	Roost-MML
Steels	0.125(−1.93)	0.982(−0.22)	0.8(0.27)
JDFT	0.078(−2.35)	**0.021(−3.86)**	0.654(−0.48)
Phonons	**0.005(−5.45)**	0.306(−1.17)	0.224(−1.43)
Dielectric	**0.006(−5.40)**	**0.002(−7.63)**	0.682(−0.44)

a We indicate the p-value followed by the t-statistic in parentheses. Statistically significant improvements are shown in boldface.

For the four data sets (<5000 samples), we found that the performance improvements achieved using Roost-SSL and Roost-FL were significant for two data sets. We note that although the Roost-MML strategy did show negative t-statistics on 3 out of the 4 data sets, suggesting improved performance in terms of MAE, the higher p-values indicate that these gains are statistically insignificant. However, these methods might prove particularly valuable in instances where the stoichiometric formula fails to capture structural differences that, in turn, lead to variations in properties. For example, with the perovskites data set, we observe a p-value of 0.01 and a t-statistic of −0.87 for the Roost-MML model indicating its usefulness in scenarios where capturing structural differences is important. Further exploration of the Roost-MML strategy, especially in cases where structure largely dictates property differences, could be a promising direction for future work.

**Figure 8:**
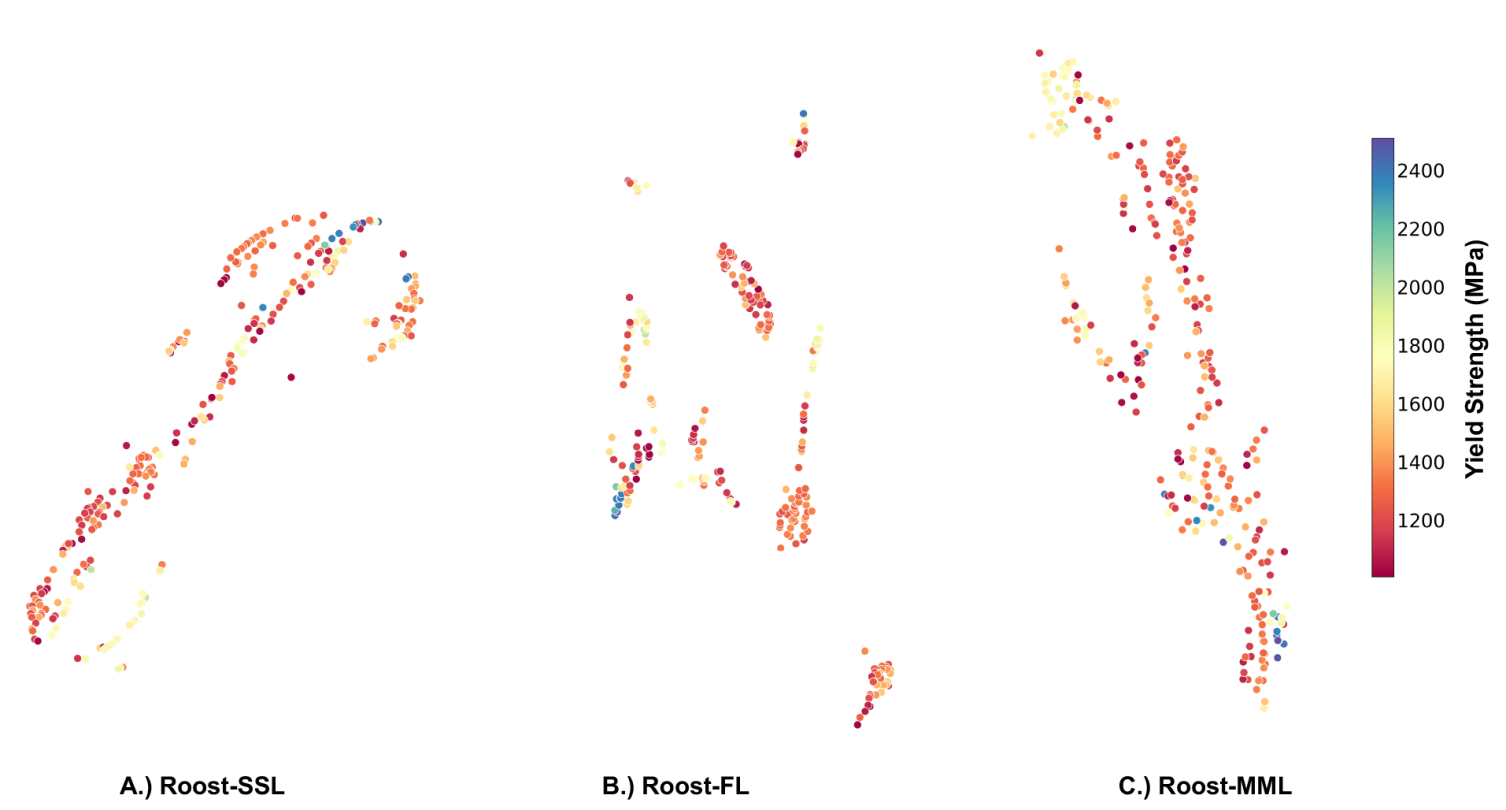
t-SNE representation for the steels data set. A.) Pretrained Roost-SSL representation for the steels data set. B.) Pretrained Roost-FL representation for the steels data set. C.) Pretrained Roost-MML representation for the steels data set.

Furthermore, we examined the pretrained representations learned by the model for the smallest steels data set using t-SNE.^55^ The t-SNE representations for the steel data set are shown in Figure 8. For the steels data set, the Roost-SSL strategy tends to group data with high yield strength to the top right of Figure 8A. In contrast, samples with lower or medium yield strength are mainly found in the lower left region. With the FL strategy, as shown in Figure 8B, steels with high yield strength cluster on the left side of the plot. And for the MML strategy, high yield strength steels are predominantly in the bottom right (Figure 8C).

### Data Efficiency

**Figure 9:**
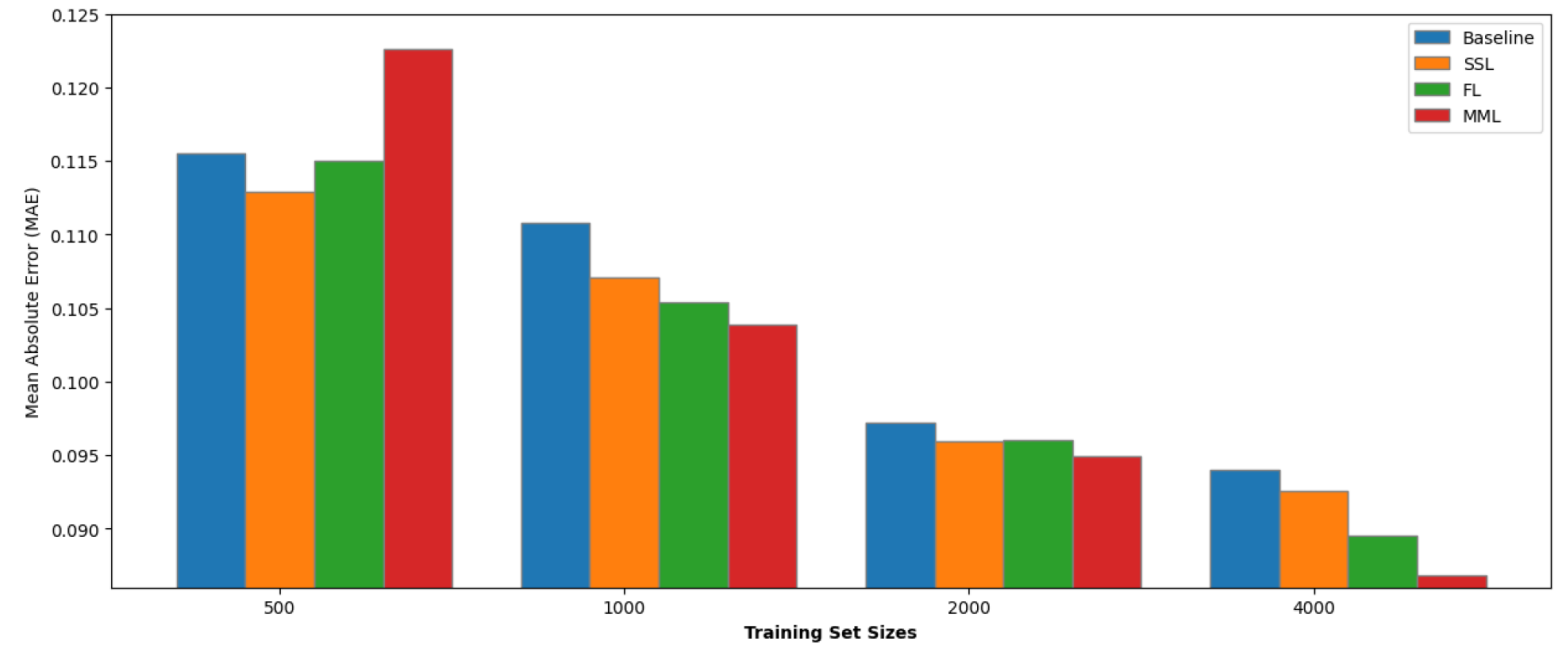
MAE of different pretrained methods for subsets of the KVRH data set.

**Figure 10:**
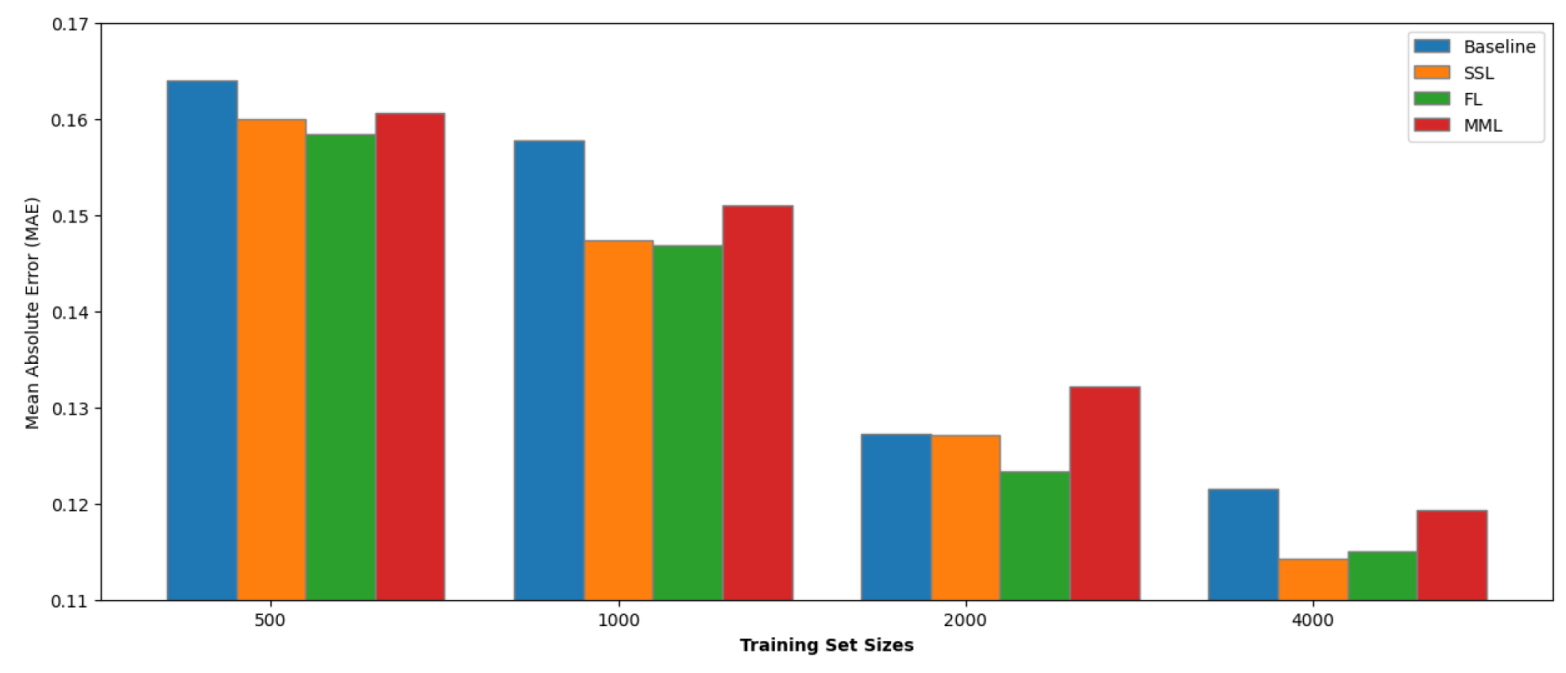
MAE of different pretrained methods for subsets of the GVRH data set.

We conducted an experimental investigation to assess how pretraining influences our model’s performance across different data set sizes. This experiment involved generating subsets of various sizes (500, 1,000, 2,000, and 4,000 samples) from our medium-sized data sets KVRH and GVRH. The histograms are shown in Figure 9 and Figure 10.

Our results presented a nuanced picture of the efficacy of pretraining strategies. SSL, FL, and MML methods improve the model performance average by 4.67%, 3.76%, and 1.05% on the GVRH data set and 2.83%, 2.10%, and 2.53% on the KVRH data set. Notably, the SSL and FL outperformed the baseline in both data sets and exhibited a consistent improvement in performance across all subset sizes.

In contrast, the MML model showed more variable performance. While it often surpassed the baseline in most subsets, there were notable instances where its performance declined. This fluctuation is hypothesized to stem from the divergence in data distribution between the pretraining and downstream task data sets, and there are fewer elements in the pretraining hMOF data set, highlighting the sensitivity of pretraining efficacy to the nature of the data used to pretrain the model.

All three methods exhibited optimal improvements when applied to data sets of approximately 1,000 samples in size. Specifically, the SSL approach enhanced performance by 6.53% and 3.34% for GVRH and KVRH properties, respectively. The FL method showed an improvement of 6.91% and 4.87%, while the MML approach resulted in gains of 4.25% and 6.22%.

A similar result was observed in the Phonons data set, which comprises 1,265 samples. It indicates the model has benefited the most at around this size. On the one hand, for data set sizes smaller than this range, the influence of pretraining, which is typically conducted on larger and more diverse data sets, may not be sufficient to fully recalibrate the model weights. Conversely, as the size of the downstream data set increases, the models gain the capability to train effectively from scratch, reaching performance levels comparable to the pretrained model. This observation also points to an inherent limitation in the diversity and quantity of the pretraining data sets; a detailed discussion is in the Supporting Information section.

## Discussion and Conclusion

In this work, we have developed and implemented three pretraining strategies specifically designed for structure-agnostic material property prediction. These included Self-Supervised Learning, Fingerprint Learning, and Multimodal Learning for pretraining the Roost encoder. The use of these pretraining strategies resulted in a noticeable improvement in the performance of downstream material property prediction tasks. Importantly, these strategies proved to be particularly effective for smaller data sets. Such data sets with limited information are often encountered in real-world scenarios. Our method’s structure-agnostic characteristic ensures adaptability, making it especially appropriate for situations with scarce data. This adaptability aids in the efficient screening of materials. While the results are promising, we acknowledge a notable limitation of structure-agnostic models: the inability to differentiate between isomers. Addressing this limitation by enabling the models to discern structural differences among isomers could lead to significant advancements in encoder performance, thereby enhancing the accuracy and reliability of material property predictions. Furthermore, our study provides pretraining strategies and opens avenues for further investigation into the nature of the pretraining data. Determining the most effective types of pretraining data and understanding how they influence downstream task performance are areas ripe for exploration. This line of inquiry is crucial, as it could provide insights into optimizing pretraining strategies for enhanced model effectiveness, especially in the context of data-limited environments. Ultimately, the positive effect observed in the performance of our models underscores the potential and effectiveness of our pretraining strategies, specifically in the context of structure-agnostic approaches. We anticipate that the pretraining techniques developed in this study can be applied to other structure-agnostic models, as well. This adaptability holds significant promise for enabling the learning of more robust and generalizable material representations that do not rely on explicit crystal structure information. By successfully navigating the challenges associated with limited data, we believe that the improvements achieved through these pretraining strategies can play a pivotal role in accelerating the material discovery process.

## Data Availability

The pretraining data used in this study was obtained from the Roost repository, which can be accessed at https://github.com/CompRhys/roost. Notably, a portion of the Roost data was sourced from the Open Quantum Materials Database (OQMD), accessible at https://oqmd.org/. The training data was acquired from Matbench, which is publicly available at https://matbench.materialsproject.org/. The code is available at https://github.com/hongshuh/PretrainRoost.

## Supporting Information Available

Additional details about the hyperparameters used for pretraining and finetuning the models, ablations done on the pretraining data set (PDF)
