# Supplementary material for: Pretraining Strategies for Structure Agnostic Material Property Prediction
Source: J Chem Inf Model. 2024 Feb 1;64(3):627–37. doi: 10.1021/acs.jcim.3c00919 (PMC10865364; doi:10.1021/acs.jcim.3c00919)
Supplement: Supplementary file 1 — ci3c00919_si_001.pdf [file ci3c00919_si_001.pdf]

Supporting Information
Pretraining Strategies for Structure Agnostic Material Property Prediction

Hongshuo Huang,[†,¶] Rishikesh Magar,[‡,¶] and Amir Barati Farimani[*,‡,†]

†*Department of Material Science and Engineering, Carnegie Mellon University, Pittsburgh PA, USA 15213*

‡*Department of Mechanical Engineering, Carnegie Mellon University, Pittsburgh PA, USA 15213*

¶*Joint First Authorship*

E-mail: barati@cmu.edu

Hyperparameters for Self-Supervised Learning(SSL)

We carefully optimized the hyperparameters to achieve the best performance in our self-supervised learning framework. We began by experimenting with different batch sizes, starting with smaller ones. We observed that the loss did not decrease for smaller batch sizes; however, for larger batch sizes, the Barlow Twins loss was able to decrease effectively. We ultimately chose a batch size of 16,384, as it demonstrated better performance in downstream tasks. Furthermore, we investigated the optimal number of epochs by evaluating the model's performance at 10, 50, and 300 epochs. We found that the loss converged around 50 epochs and continued to decrease slowly beyond that point. However, when comparing the results in downstream tasks, the model trained for 50 epochs exhibited the best performance. Con-

sequently, we finalized our hyperparameter settings with a batch size of 16,384 and training for 50 epochs. We also experimented with validation ratios of 0.01 and 0.05 during our model optimization. Although the validation loss was lower with a ratio of 0.01 during pretraining, better performance was observed in downstream tasks using a ratio of 0.05. A key takeaway is that setting the validation ratio too low could compromise the effectiveness in downstream tasks.

Additionally, we explored varying the embedding size used in the Barlow Twins loss. While the original paper suggests that larger embedding sizes lead to better performance, but larger embedding sizes demanded higher GPU memory, which was a limiting factor in our experiments. Taking these considerations into account, we decided to set the embedding size at 1024 for our model. The hyperparameters used for SSL pretraining are shown in Table S1.

Table S1: Hyperparameters for pretraning SSL

Hyperparameters	Value
Epochs	50
Batch Size	16384
Learning Rate	0.0001
Optimizer	Adam
Validation Ratio	0.05
Pretrain Data Size	432k
Embedding Size	1024
λ	0.0051

Hyperparameters for Fingerprint Learning(FL)

To ensure that the Roost embeddings closely resemble the Magpie fingerprint, we aim to minimize the mean squared error (MSE) loss using gradient descent. This process requires all values to be numerical. However, one of the features, ”compound possible,” is a Boolean variable. As a result, we decided to exclude this feature from our analysis.

Furthermore, it is essential to normalize all values to ensure equal contribution towards

approximating the fingerprint and to prevent the model from being biased by features with large values. After normalizing the remaining 144 Magpie features, we use them as the fingerprint for our Fingerprint-Learning framework. The hyperparameters for FL are shown in Table S2. The hyperparameters for FL were aligned with the SSL strategy, except for adjustments in the batch size and the number of epochs. We determined the number of epochs by observing the point where the loss no longer decreased. Pretraining was done for 100 epochs using this strategy. The batch size was optimized based on the memory constraint of the GPU.

Table S2: Hyperparameters for FL

Hyperparameters	Value
Epochs	100
Batch Size	256
Learning Rate	0.0001
Optimizer	Adam
Validation Ratio	0.05
Loss	MSE
Pretrain Data Size	432k

Hyperparameters for Multimodal Learning(MML)

In this study, we leverage the CGCNN backbone, obtained from the Crystal Twins framework,[1] to generate embeddings for the hMOF dataset. We then utilize a structure-agnostic model to predict these embeddings. The hyperparameters for the MML model are shown in Table S3. The hyperparameters for MML were kept similar with the other pretraining strategies, with the exception of batch size and the number of epochs. We increased the number of epochs because we observed that the pretraining loss decreased and plateaued after 90 epochs. Consequently, training was terminated at 100 epochs. The batch size was optimized based on the memory constraint of the GPU.

Table S3: Hyperparameters for MML

Hyperparameters	Value
Epochs	100
Batch Size	256
Learning Rate	0.0001
Optimizer	Adam
Validation Ratio	0.05
Loss	MSE
Pretrain Data Size	137,652
Embedding Size	256

Details about the Pretraining dataset

Data quality

The quality of pretraining data has a direct impact on the representations learned by the model. In the case of SSL for images, noisy images can lead to the learning of misleading features. Similarly, for materials property prediction, certain materials with only one or two elements may present challenges. After masking one node in such materials, they could lose 50% or even 100% of their node features, which may cause the model to learn irrelevant features.

Data diversity and Quantity

A diverse pretraining dataset, encompassing various examples from the domain of interest, allows the SSL model to learn generalizable features that can be applied to different downstream tasks. The pretraining dataset should including a wide range of materials, such as metals, ceramics, polymers, composites, perovskites, and others, in order to provide the model with a comprehensive view of the materials landscape. If the pretraining data is limited to a specific class of materials, like metals, the model may become biased towards patterns specific to that class, hindering its ability to generalize to other materials property prediction tasks.

To identify the most appropriate pretraining data for downstream tasks, we conducted

experiments using a variety of dataset combinations. These datasets primarily consisted of data from the Roost paper(OQMD and experimental band gap of non-metal materials), MatBench datasets, and MOF (Metal-Organic Framework) data. The results is shown in Table S4. It is observed that unique materials from all datasets have the highest improvement in performance for the materials property prediction tasks since it have the greatest quantity and diversity of data. This ensures the model learns robust and generalizable features.

Data diversity and quantity of dataset are highly correlated. We also examine how the size of pretraining dataset impact the downstream performance. If the pretraining dataset contains a small number of samples, the model during pretraining may not learn to capture the complex relationships in materials. To this end, we examine the influence of availability of pretraining data for the Roost model. We define the datasets used in the Roost paper[2] as Roost data. The aggregation of the all the datasets from the Matbench suite as Matbench data[3] and hMOF database as MOF data.[4] The results of our experimentation for pretraining datasets is shown in Table S4

Table S4: Average Improvement on 9 matbench dataset for different pretraining dataset size

Pretraining Data(K)	300[1]	369[2]	405[3]	413[4]	420[5]	432[6]
Average Improvement(%)	2.0	0.24	3.66	4.20	2.85	7.65

[1]Roost Data
[2]Unique (Roost data + Matbench data)
[3]Unique Roost data + Unique Matbench data - Perovskites
[4]Unique Roost data + Unique Matbench data
[5]Unique Roost data + Unique Matbench data + Unique MOF data
[6]Unique (Roost data + Matbench data + MOF data)

Performance Improvements after Finetuning

We evaluate performance improvements compared to the baseline Roost[2] model. We observe that pretraining with SSL strategy is the most effective. Impressive gains are observed for small datasets and medium sized datasets for both SSL and FL strategy. The SSL and FL strategies are unable to improve the performance on larger dataset when compared

to supervised learning. The MML strategy shows gains on the larger datasets, probably because the effects of learning structure features is most prominent for larger datasets. The performance gains are shown in Table S5. The mp-non-metals dataset is a subset of non-metals materials in the mp-gap dataset. Additionally, we also investigate the significance of these improvements for the SSL strategy.

Table S5: Improvement in downstream tasks performance compared to the supervised Roost model

Dataset	SSL-Roost	FL-Roost	MML-Roost
jdft2d[5]	4.06%	4.45%	-0.79%
phonons[6]	15.33%	4.53%	1.94%
dielectric[7]	4.00%	2.644%	1.00%
log-gvrh[8]	2.75%	-1.10%	-0.17%
log-kvrh[8]	2.69%	2.66%	1.83%
perovskites[9]	-0.63%	-0.45%	0.28%
mp-gap[10]	-2.94%	0.41%	0.79%
mp-e-form[10]	-0.87%	0.40%	1.45%
steels	14.88%	0.24%	3.33%
average	3.64%	1.53%	1.07%

Hyperparameters for Finetuning on Matbench datasets

We evaluate the performance of pretrained models on the Matbench suite. The Table S6 shows the hyperparameters that we use to finteune the roost encoder for the matbench suite. We would like to note that the same hyperparameters were used for all datasets.

Table S6: Hyperparameters for Finetune

Hyperparameters	Value
Epochs	[250,100[1]]
Batch Size	128
Learning Rate	3e-4
Optimizer	AdamW
momentum	0.9
weight decay	1e-6
element feature	64
n graph	3
Loss	L1 Robust loss
ensemble	1

[1]Perovskites dataset

References

(1) Magar, R.; Wang, Y.; Farimani, A. Crystal twins: self-supervised learning for crystalline material property prediction. npj Comput. *Mater* **2022**, *8*, 231.

(2) Goodall, R. E.; Lee, A. A. Predicting materials properties without crystal structure: Deep representation learning from stoichiometry. *Nature Communications* **2020**, *11*, 1–9.

(3) Dunn, A.; Wang, Q.; Ganose, A.; Dopp, D.; Jain, A. Benchmarking materials property prediction methods: the Matbench test set and Automatminer reference algorithm. *npj Computational Materials* **2020**, *6*, 1–10.

(4) Wilmer, C. E.; Leaf, M.; Lee, C. Y.; Farha, O. K.; Hauser, B. G.; Hupp, J. T.; Snurr, R. Q. Large-scale screening of hypothetical metal–organic frameworks. *Nature chemistry* **2012**, *4*, 83–89.

(5) Choudhary, K.; Kalish, I.; Beams, R.; Tavazza, F. High-throughput identification and characterization of two-dimensional materials using density functional theory. *Scientific Reports* **2017**, *7*, 1–16.

(6) Petretto, G.; Dwaraknath, S.; PC Miranda, H.; Winston, D.; Giantomassi, M.; Van Set-

ten, M. J.; Gonze, X.; Persson, K. A.; Hautier, G.; Rignanese, G.-M. High-throughput density-functional perturbation theory phonons for inorganic materials. *Scientific data* **2018**, *5*, 1–12.

(7) Petousis, I.; Mrdjenovich, D.; Ballouz, E.; Liu, M.; Winston, D.; Chen, W.; Graf, T.; Schladt, T. D.; Persson, K. A.; Prinz, F. B. High-throughput screening of inorganic compounds for the discovery of novel dielectric and optical materials. *Scientific data* **2017**, *4*, 1–12.

(8) de Jong, M.; Chen, W.; Angsten, T.; Jain, A.; Notestine, R.; Gamst, A.; Sluiter, M.; Krishna Ande, C.; van der Zwaag, S.; Plata, J. J.; Toher, C.; Curtarolo, S.; Ceder, G.; Persson, K. A.; Asta, M. Charting the complete elastic properties of inorganic crystalline compounds. *Scientific Data* **2015**, *2*, 150009.

(9) Castelli, I. E.; Olsen, T.; Datta, S.; Landis, D. D.; Dahl, S.; Thygesen, K. S.; Jacobsen, K. W. Computational screening of perovskite metal oxides for optimal solar light capture. *Energy Environ. Sci.* **2012**, *5*, 5814–5819.

(10) Jain, A.; Ong, S. P.; Hautier, G.; Chen, W.; Richards, W. D.; Dacek, S.; Cholia, S.; Gunter, D.; Skinner, D.; Ceder, G.; Persson, K. A. Commentary: The Materials Project: A materials genome approach to accelerating materials innovation. *APL Materials* **2013**, *1*, 011002.
